# Rebiasing: Managing automatic biases over time

**DOI:** 10.3389/fpsyg.2022.914174

**Published:** 2022-09-29

**Authors:** Aleksey Korniychuk, Eric Luis Uhlmann

**Affiliations:** ^1^Copenhagen Business School, Strategy and Innovation, Frederiksberg, Denmark; ^2^INSEAD, Organisational Behaviour Area, Singapore, Singapore

**Keywords:** automatic evaluations, automatic preferences, biases, adaptiveness, intuition, debiasing

## Abstract

Automatic preferences can influence a decision maker’s choice before any relevant or meaningful information is available. We account for this element of human cognition in a computational model of problem solving that involves active trial and error and show that automatic biases are not just a beneficial or detrimental property: they are a tool that, if properly managed over time, can give rise to superior performance. In particular, automatic preferences are beneficial early on and detrimental at later stages. What is more, additional value can be generated by a timely *rebiasing*, i.e., a calculated reversal of the initial automatic preference. Remarkably, rebiasing can dominate not only debiasing (i.e., eliminating the bias) but also continuously unbiased decision making. This research contributes to the debate on the adaptiveness of automatic and intuitive biases, which has centered primarily on one-shot controlled laboratory experiments, by simulating outcomes across extended time spans. We also illustrate the value of the novel intervention of adopting the opposite automatic preference—something organizations can readily achieve by changing key decision makers—as opposed to attempting to correct for or simply accepting the ubiquity of such biases.

## Introduction

Decision making in organizations is prone to the effects of intuitive thinking, most notably biases (Khatri and Ng, 2000; Kahneman, 2003; Miller and Ireland, 2005). Existing work in the organizational sciences and social-cognitive psychology often focuses on debiasing interventions, in other words strategies to remove automatic biases from organizational choices (Schwenk, 1986; Wilson and Brekke, 1994; Wilson et al., 2000; Winter et al., 2007; Christensen and Knudsen, 2010). However, we show that dynamically rebiasing—that is, reversing biases by periodically adopting the opposite automatic preference—can be a strictly dominant strategy. To do so, we extend the standard model of boundedly rational search with a first principle of biased decision-making—namely, the presence of spontaneous, intuitive thinking.

Social-cognitive psychology has highlighted the layered nature of the human mind, where decision making involves the functioning of both controlled (System 2) and automatic (System 1) processes (Simon, 1990; Sloman, 1996; Stanovich and West, 2000; Newell and Simon, 2007; Evans, 2008; Evans and Stanovich, 2013). The former is the kind of thought process that comes with an effort: it is deliberate, slow, and self-aware. The latter, conversely, is the kind of thinking that we can only barely control or shape logically: it is fast, associative, and effortless (Stanovich and West, 2000). This intuitive component represents an important element of human judgment. Even in organizations, decision makers routinely call on their intuitions or “gut feelings” when making both day-to-day and long term strategic choices (Khatri and Ng, 2000; Miller and Ireland, 2005). But the effect of intuitive thinking on organizational choices is not always positive and indeed can be detrimental (Kahneman, 2003; Inbar et al., 2010). This has to do with the fact that a key aspect of effortless information processing is our ability or propensity to make automatic evaluations before perceiving complete or even meaningful information (Zajonc, 1980; Wilson and Brekke, 1994; Duckworth et al., 2002; Kahneman, 2003; Volz and von Cramon, 2006). Naturally, such reliance on arbitrary, immediately observable stimuli often results in biases, or deviations from what would be deemed appropriate by the more logical rules of System 2 (Kahneman, 2003).

Biased judgments are commonplace and have been documented in a wide spectrum of settings (e.g., Kramer et al., 1993; Stone, 1994; Nickerson, 1998; Raghubir and Valenzuela, 2006; Scott and Brown, 2006). However, despite their definitional conflict with the rule of logic in observable outcomes, beyond the scope of a single choice, biases may be beneficial (Arkes, 1991; Marshall et al., 2013). Cognitive processes of System 1 generate responses so efficiently that the organisms possessing them can have evolutionary advantages (Gigerenzer and Todd, 1999). Similarly, such responses may reflect the properties of the environments in which our intelligence has evolved (e.g., Haselton and Nettle, 2006; Johnson and Fowler, 2011). If a certain behavioral response confers propagation or survival advantages, it is more likely to be prevalent in the population long-term (Haselton and Nettle, 2006). Consequently, the positive effects of our less controlled cognitive processes and corresponding biases may only emerge over a sequence of choices and would not be captured in single-session experiments in laboratory settings.

Guided by this premise, we conjecture that positive or negative effects of cognitive manipulations (such as eliminating or altering biases) should likewise manifest themselves over a sequence of adaptive choices. Accordingly, we design a computational model of adaptive sequential trial and error that incorporates the first principles of human thinking and thus allows for a study of temporal effects of System 1 biases as well as interventions to eliminate or alter them.

We find that the consequences of biased judgments are indeed time-variant. System 1 automatic evaluations offer short-term benefits that will tend to propagate in dynamic environments that remain stable only for a limited time. However, these benefits quickly disappear, causing profound long-term harm. The reason for the observed pattern is that automatic evaluations constrain the space of options for trial and error (e.g., pick only green, no red), thereby suppressing experimentation. Further analysis of this effect reveals that manipulations of biases can offer advantages in settings with more available time. However, contrary to what may be expected, it is not debiasing (or eliminating the bias) that betters both biased and unbiased decision making, it is rebiasing (or reversing the bias). To be effective, rebiasing must take place at a calculated moment in time. An advantage, therefore, may come not from eliminating biases but from effectively managing them. Unlike individuals, organizations can in principle reverse their biases by appointing different decision makers to key roles such as top leadership positions.

## Theoretical background

Consider the following problem. A decision maker is faced with a set of options, each with a different payoff or score. These can represent monetary outcomes such as profit, or different measures of performance, for example, product quality, cost, or customer satisfaction. The goal is to discover options with greater scores (see, for example, Simon, 1955).

For a flawless intelligence, a problem like this is trivial. An omnipotent mind would immediately select the best option. Assuming that there are no information processing constraints, the number of possibilities is finite, and there are no impediments to choice, such behavior is rational. Indeed, in some situations, this kind of intelligent choice is a good proxy of that of humans. Think, for example, about choosing the biggest apple on a plate. The color, size, and shape are all directly observable and the choosing of the most appealing apple is not a problem. Given comprehensible information about all options, we simply pick the best one. However, the situation changes when we cannot process the entire set of possibilities or face noisy signals. Finding the biggest apple in a loaded trailer will already reveal the limits of our capacities.

In the middle of the last century, Herbert Simon postulated that in problems like the one above, human rationality is bounded (Simon, 1955, 1956). Instead of optimizing over the entire space of possibilities, we search and satisfice. That is, we sequentially generate and try new options until we find one that meets all essential criteria or as long as our outcomes are below aspirations (Simon, 1955; Levinthal and March, 1981; Lant, 1992). In other words, boundedly rational decision makers continuously search for better options. This model of decision making represents the kind of “behavior that is compatible with the access to information and the computational capacities that are actually possessed by organisms” (Simon, 1955, p. 99).

However, while certainly compatible with a limited intelligence, including that of a human, the Simonian representation of problem solving is not specifically human (or more broadly, biological). In particular, it omits biases that are typical of human cognition (see Fiori, 2011). The existing literature identifies a wide spectrum of intuitive biases or spontaneous “response[s] because of mental processing that is unconscious or uncontrollable” (Wilson and Brekke, 1994, p. 117). These biases systematically contaminate decision making, often without the person’s awareness of their influence. Indeed, such blindness to the rationale behind one’s own choices reflects the complexity of human thought (Nisbett and Wilson, 1977; Greenwald and Banaji, 1995; Haidt, 2001; Kahneman et al., 2011).

Extensive research in psychology indicates that human cognition involves the simultaneous functioning of two systems (Sloman, 1996; Kahneman, 2003). One system (System 1) is spontaneous, intuitive, uncontrolled, and fast—this system is based on the law of association. The other system (System 2) is deliberate, effortful and relatively slow—this system can be said to rely on the law of logic (Stanovich and West, 2000). However, the responses of these systems to exogenous stimuli do not always align. In situations in which System 1 dominates System 2 (e.g., limited time, high cognitive load, or when the choice is closer to perception than to deliberate assessment), the decision maker’s judgment is especially likely to deviate from the rules of logic (Fazio, 2001). Although there are exceptions, such as expert intuition trained in repetitive and predictable settings—think about chess (Kahneman and Klein, 2009)—in real-world situations automatic evaluations will not always be “reasonable by the cooler criteria of reflective reasoning. In other words, the preferences of System 1 are not necessarily consistent with preferences of System 2” (Kahneman, 2003, p. 1463). This inconsistency can take multiple forms but fundamentally it reduces to an arbitrary preference for a certain, immediately observable or perceivable attribute of options (Zajonc, 1980; Fazio et al., 1986; Fazio, 2001; Duckworth et al., 2002; Slovic et al., 2002).

Such preferences form as a part of automatic evaluations that do not require conscious reasoning and occur even when the stimuli are novel (Zajonc, 1980; Fazio et al., 1986; Greenwald and Banaji, 1995; Fazio, 2001; Duckworth et al., 2002). While these affective responses are variegated (Hutchinson and Gigerenzer, 2005), in the context of choice, they fundamentally reduce to a form of heuristic that accepts or rejects based on a certain immediately perceivable attribute of options. That is, “pick A, if A is” more readily accessible, more representative of a category, implies lesser losses, etc.

To the extent that this immediately observable attribute is uncorrelated with the target criterion (i.e., the performance score, quality, cost, etc.), the ultimate choice will be subject to biases. Importantly, the presence of these biases is not uniform over all stages of the decision-making processes. Specifically, the greater the involvement of System 1, the more liable to biases the choice is. This happens because intuitive judgments originate “between the automatic parallel operations of perception and the controlled serial operations of reasoning” (Kahneman and Frederick, 2002, p. 50). Somewhere between perception and more deliberate processes of reasoning, a human-like intelligence will have a quick, spontaneous evaluative response that may direct the ultimate choice (Zajonc, 1980; Kahneman, 2003).

Existing experimental studies have shown that biases appear in a wide variety of trivial choices (Tversky and Kahneman, 1974). A natural consequence is that biases permeate human and by extension organizational decision making. This, in turn, can hold implications for organizational performance. Accordingly, scholars have analyzed the role of biases from various organizational perspectives, from their effects on strategic decision making (Schwenk, 1984, 1986; Lyles and Thomas, 1988; Reitzig and Sorenson, 2013) to their implications for organizational adaptation (Denrell and March, 2001). However, in this stream of work, biases have been essentially equated with some form of evaluation imperfections and thus no different from systematic errors in deliberate decisions. The automatic, spontaneous nature of the underlying cognitive processes remains largely unintegrated with boundedly rational problem solving at the individual or organizational levels. This omission limits our understanding of how organizations can leverage the idiosyncrasies of human decision making.

In the following section, we develop a parsimonious model of boundedly rational problem solving with unreasoned automatic evaluations (i.e., automatic biases). We then use this model to illustrate the temporal consequences of intervening to eliminate or change biases. Our work specifically assesses the effectiveness of two basic strategies that organizations can use to manipulate biases: de-biasing, or entirely eliminating a bias, and re-biasing, or adopting the exact opposite automatic preference, as well as their optimal timing.

## Model setup and analyses

**FIGURE 1:**
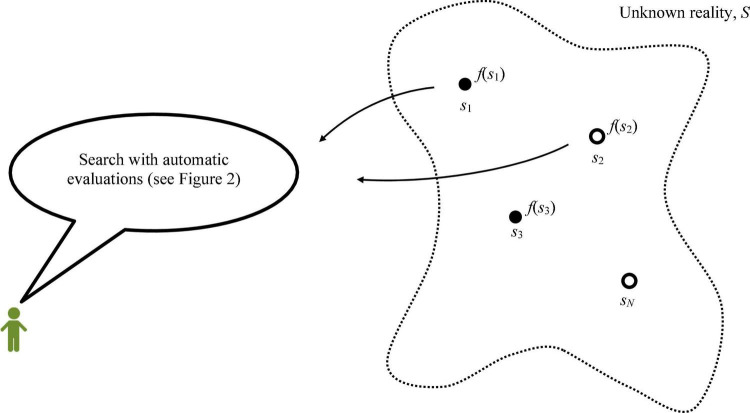
Problem illustration. The objective is to find option *s*_*n*_ with the highest score, f. The immediately observable attribute *ξ* is represented by whether each option is black or white. The true score *f*(*s*_*n*_) is known only upon trial.

Our model has two basic elements: (i) an unknown reality with *N* options, (ii) a process of search that proxies problem solving by a boundedly rational intelligence with automatic evaluations. Figure 1 illustrates these elements.

### Unknown reality

Reality is represented by a set of options, *S*, where each option *s*_*n*_ has two attributes. For a trivial example, consider a bucket of exotic fruits. Let’s call them *karamzamsas*. The first attribute, *ξ*, is an immediately perceivable property, e.g., size, color, smell, etc. of a *karamzamsa*. We assume this attribute to take on one of two values, 0 or 1, i.e., *ξ*∼ *U*{0, 1}. The second attribute, *f*, represents the true value of the option, e.g., taste, nutritional content, etc. Without loss of generality, we assume that this value is distributed normally, i.e., *f*(*s*_*n*_) ∼ *N*(0, 1). The true value of each option is observable only upon trial. That is, to know how a *karamzamsa* tastes, we need to take a bite.

### Search with automatic evaluations

Consistent with the first principles of bounded rationality, our agents sequentially generate and try new options. However, we consider that although able to try only a single option at a time, agents can perceive multiple possibilities simultaneously. This is a key distinctive element of our conceptualization: at every moment in time, agents simultaneously perceive multiple options, but can try or experience only a single one. Continuing our example with a bucket of *karamzamsas*, consider that these exotic fruits are small and we can hold several of them in one hand. So we grab a handful and then drop all but the one we want to taste. For a more practical analogy, think about serial entrepreneurs or startups that come up with various business ideas but implement only a single one at a time. For an analogy that closely maps onto the underlying assumptions, think about the many choices organizational executives make on a daily basis: appointing the right subordinates, selecting suppliers, discontinuing products, etc.^1^ In many ways, these decisions are logically equivalent to exotic fruits: there is a multitude of them and their value, like that of *karamzamsas*, becomes fully identified only upon trial.

With this basic setup, we can understand the effect of biases that come with automatic evaluations. Unbiased agents will automatically select a random option. Think about a person who has never tried any fruit. This person will not be able to tell *karamzamsas* apart: a green *karamzamsa* looks just as good as a red one. On the contrary, a person who is fond of red apples, may automatically select red *karamzamsas*. Green *karamzamsas* are, of course, as good as red *karamzamsas*. But the person who likes red apples will tend to pick red *karamzamsas*. This is the logic of a biased agent, an agent with automatic evaluations who exhibits systematic preferences for an irrelevant immediately observable attribute of options. Although in the case of *karamzamsas*, such a bias will likely quickly disappear as the agent learns about the true taste of these wonderful fruits, many real-world biases are hard to eradicate even given the agent’s full awareness (Wilson and Brekke, 1994). Such persistent biases in our automatic evaluations will interplay with our problem solving long-term.

**FIGURE 2:**
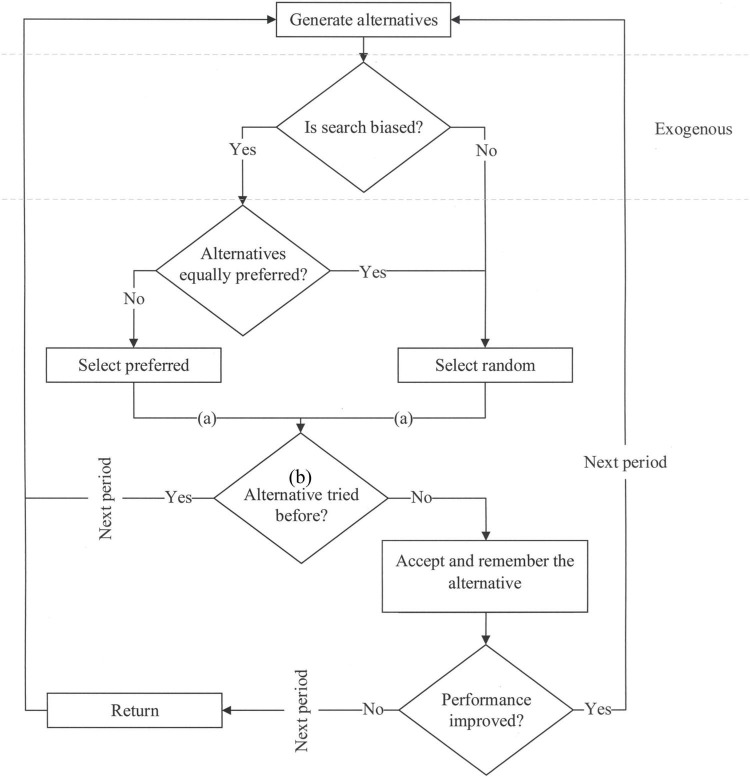
Search with automatic evaluations. The letters indicate the following: (a) the end of System 1 information processing; (b) agents deliberately assess, i.e., compare to previous trials, one alternative per period.

Similar to Jung et al. (2021) we illustrate the logic of the search process with an algorithm. However, our algorithm does not have a defined stopping point. This implies that the agents continuously adjust their aspirations and continue searching for better solutions. Figure 2 illustrates this algorithm and the distinction between the two categorical extremes, biased and unbiased search, in stricter terms. Unbiased search approximates problem solving of a bounded intelligence that has no automatic evaluations. Biased search is a proxy for a human-like intelligence that exhibits automatic evaluations. If the search is biased, the agents will effectively reject options based on the irrelevant criterion *ξ* every time they simultaneously perceive an option they prefer.

The logic of the algorithm is as follows. Generate or perceive several options. If one of these options dominates other options in terms of the immediately observable criterion *ξ*, select this option for thorough consideration and trial. If the selected option has been tried before, disregard it and restart the process of search. If the selected option has not been tried before, try it and observe its performance. We measure performance as the value *f*(*s*_*n*_) of the currently accepted option. If the performance improves, i.e., if *f*(*s*_*t*_) > *f*(*s*_*t*–1_), where *t* indicates the moment in time, accept this option, i.e., *f*(*s*_*t*_), as a new *status quo*. If the performance declines, i.e., if *f*(*s*_*t*_) < *f*(*s*_*t*–1_), continue to the next period and when it starts remember to return to the *status quo*, or the best option discovered thus far, i.e., *f*(*s*_*t*–1_).

With this algorithm, we run a simulation model. In particular, we create a random set *S* of 100 options,^2^ and assume that the agents sample options from this set with replacement. In every period, an agent generates two random alternatives from set *S*, picks one of the two generated options following the biased or unbiased process and then either tries this option or moves to the next period (see Figure 2). Our observations are averaged over at least 10^6^ simulations. This amount of simulations ensures that the reported patterns are stable and reproduce with near certainty. Simulations were coded in Code:Blocks 16.01 in C++ programming language following C++ 11 ISO standard. The complete data and code are posted on the Open Science Framework at https://osf.io/sypn2/?view_only=1b00c0d2dc964bafadf10215bfca4743.

Before we proceed to our observations, let us make some important clarifications and caveats. First, the process, where the tried option can be sampled repeatedly, proxies a situation with a multiplicity of similar choices that have the same performance. To see what this means in the context of organizational decision making, consider, for example, a situation where a company from the capital region of Denmark unsuccessfully expands to the rest of the country. If establishing operations in Aalborg was not successful then probably (for the sake of argument, consider that these two cities are sufficiently similar along the dimensions relevant for the organizational offer) it will also fail in Odense. Then, if after a failure in Aalborg, decision makers come up with the idea of starting operations in Odense, they will effectively have generated the same option again. This, of course, is only a hypothetical illustrative example. Possibilities vary (e.g., smaller cities in Denmark like Roskilde or Ringsted may turn out to represent a different option). The logic of the model is, of course, agnostic to the exact criterion. Sampling with replacement captures only the idea that some similar options have the same performance and can be intuitively generated or perceived separately.

Second, given the example above, a careful reader may wonder whether it is appropriate to compare an expansion to Aalborg in, for example, 2010 with an expansion to Odense in say 2035. Probably not. In fact, it may be equally unjustified to compare Aalborg in 2010 and Aalborg in 2035. The social, environmental, market, and even political conditions may be completely unalike. For this reason, time is a critical variable in our analysis because we compare performance in solving a given problem. The problem, of course, remains the same as long as the set of options *S* is constant. A meaningful change in the composition of this set, however, will essentially mean that the agents start solving another problem and the clock should start anew. Evolution of the problem, i.e., a gradual change in the composition of the set *S*, is another possibility. In the interest of clarity, we leave these issues beyond the scope of the present study and focus on the temporal effects of automatic biases when solving a given problem. That is, our agents search a fixed set of possibilities *S* and we observe their performance over time, i.e., the number of sequential choices made.

Finally, as any analytical tool, our model has boundary conditions. Our analysis captures a specific task environment designed to reflect the essential basics of many decision making situations. Although properties of this task environment are arguably general and sufficient for the following effects to hold in some other contexts of interest, the characteristics and complexities of specific real-world situations may differ and the model does not necessarily bear on them. These properties of the model can be summarized as follows: each option is characterized by two variables, one of which is directly observable and the other requires at least partial testing; decision makers are biased with respect to the observable variable but have no bias with respect to the unobservable variable of interest; the bias with respect to the observable variable materializes before any testing of the observable variable can be performed; and the two variables do not correlate with each other. The more overlapping features between the real situation and the simulated one, the more the simulation is relevant. The core code for our analyses is publicly posted, and we encourage the scientific community to explore alternative parameters more closely aligned with their specific decision making environments of interest.

### The basic effect

**FIGURE 3:**
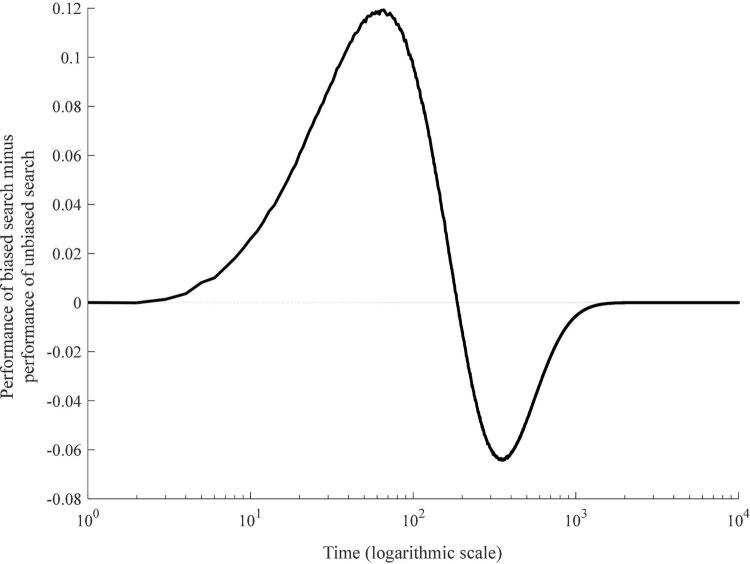
Performance of biased search relative to unbiased search.

Figure 3 shows the relative effect of biased search. Positive (negative) values indicate that at the given moment in time, the biased agent has an advantage (disadvantage) over the unbiased agent. The value of zero means that biased and unbiased agents tend to have exactly the same performance.

An immediate observation is that the effect of automatic evaluations is time-variant. System 1 biases are beneficial in the short-term and yet harmful in the long run. Note that the model timings have no direct correspondence to real-world time. The model time is measured in terms of the number of steps or decisions made or, equivalently, the number of options considered for trial. A few steps (decisions) into the process of search, automatic evaluations can generate better performance by up to ∼0.12 scores or 27% of the absolute performance of unbiased agents. Note that the magnitude of the advantage in terms of percentage peaks earlier. Early in the process of search, the absolute performance is relatively low and thus, every additional score represents a greater portion. Consider that 65 steps into the process of search, the benefit of biased search equals 0.1192 scores or 11.4% of 1.045 scores gained at that point by the unbiased agent. On the contrary, 5 steps into the process of search, the benefit of biased search is only 0.008163 scores. But in percentage terms, this represents 27.21% of 0.03 scores gained by the unbiased agent at that time. This advantage, however, is relatively short-lived. Already 187 steps into the process of search, biases become detrimental. Although the magnitude of this effect does not exceed 2.7%, it continues (albeit monotonically declining) until the problem is solved, at which point biased and unbiased agents find the best alternative and their performances converge.

### The mechanism

To understand the reasons for the observed pattern, consider what happens as the agents search the set of possibilities *S*. Every time the agents try a new option, their expected performance is 0. Recall that since *f*(*s*_*n*_) ∼ *N*(0, 1), *E*[*f*(*s*_*n*_)] = 0. The difference between their *status quo* and the expected performance is essentially the implicit cost of experimentation. As long as their performance is greater than 0, every time they try a new option, their performance will fall until they return to the *status quo*. However, sometimes it will rise and their new *status quo* will improve measurably. This is how the agents learn, i.e., increase their accumulated knowledge about the problem.

**FIGURE 4:**
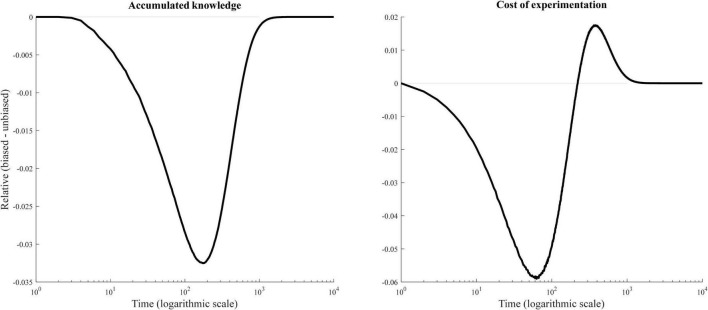
Mechanisms.

Accordingly, the effect in Figure 3 is a product of two processes (see Figure 4). First, automatic evaluations direct agents to the options they prefer (i.e., are biased toward). As a result, a biased agent learns less, i.e., accumulated knowledge is lower, because it repeatedly draws from the same subset of possibilities. In contrast, an unbiased decision maker does not rely on automatic evaluations and therefore faces lower redundancies in learning.

However, there is a second process. Learning about the problem requires experimentation, and experimentation is costly. Automatic evaluations make it less likely that the agents try new options and thereby regulate the excess of experimentation in the initial phase of problem solving. Early in the process of search, there is little knowledge about the set of possibilities *S*, which means that there are plenty of unknown options, each of which has an expected performance of 0. The probability of trying new options is very high during this time. Automatic evaluations reduce this probability and thereby increase the value from stability. Over time, this value declines as the agents learn about the problem. Past experience with a given option helps resolve uncertainty about its potential: agents know that such an option is inferior to their *status quo* and therefore need not try it.

The curves in Figure 4 illustrate the dynamics of accumulated knowledge and the implicit cost of experimentation in relative terms, where zero means that there is no difference between biased and unbiased agents. The left panel shows the dynamics of accumulated knowledge. We measure accumulated knowledge as the score of the best option known to the agent. The right panel shows the cost of experimentation. We measure the cost of experimentation as the probability of trying a new option.

### Rebiased and debiased search

In our analyses above, we assumed that biases remain constant during the entire process of search. While this is often the case, biases need not persist unchanged. Automatic evaluations exhibit high degrees of variability across people, such that different individuals can have idiosyncratic and atypical biases (Fazio et al., 1986; Baron, 2000). This variability may be used to change biases without altering the encoded memory or association. Teams, organizations, and societies can replace key decision makers with others who are less biased or hold different biases. Case studies highlight instances in which companies have changed management teams and completely reversed their previous management practice orientations (see for example, Maddux et al., 2014). At the individual level, various psychological techniques, such as framing, may activate different automatic associations and thus elicit different automatic preferences or biases within the same person (Kühberger, 1998; Chong and Druckman, 2007). Scholars in psychology as well as industry practitioners have discussed an array of techniques that can abate the effect of biases, or debias, decision making (see Kahneman et al., 2011). Similarly, the literature in management has shown that organizations have structural means to manipulate and attempt to reduce bias in organizational decision making (see Christensen and Knudsen, 2010).

Accordingly, we examine temporal implications of two interventions or manipulations of bias: rebiasing (changing the bias to its opposite), and debiasing (eliminating the bias entirely). We operationalize rebiasing as adopting the exact opposite of the initial bias, i.e., pick red instead of green, when previously the automatic preferences was green over red. Debiasing means the agent no longer relies on any irrelevant signal. Consider our example with the exotic fruit *karamzamsa* and suppose that this fruit comes in two colors: red and green. As before, both green and red *karamzamsas* are equally tasty. Then, if our decision maker prefers red apples, this decision maker will likely favor red *karamzamsas*. Rebiasing in this case would be to now have a decision maker who prefers green apples. By analogy, debiasing would mean having a decision maker who equally prefers red and green apples. We are agnostic as to the exact levers that organizations or collectives use to manipulate biases—whether they involve replacement of the key decision makers or implementation of other management practices—and focus solely on the outcomes of such strategic interventions. Our starting condition is that of the biased firm and its performance dynamics. Subsequently, we examine the temporal implications of rebiasing and debiasing.

**FIGURE 5:**
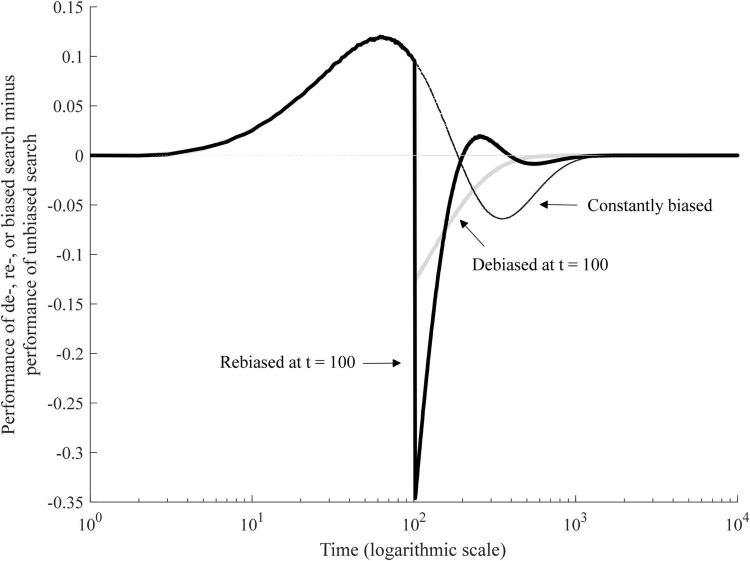
Rebiased, debiased, and constantly biased search compared to unbiased search.

Figure 5 shows the effects of these manipulations. The curves show relative performance of debiased and rebiased search (cf. Figure 3). The value of zero indicates that the difference between unbiased and debiased or rebiased agents is nil.

Contrary to what might be expected, debiasing does not result in simple convergence with unbiased search. Immediately after debiasing, there is a sharp decline in performance (see Figure 5). This happens because the set of options that used to be intuitively discarded remains comparatively unknown. So, when the bias disappears, the likelihood of trying new options goes up, which in turn increases the cost of experimentation. However, since a large portion of the possibilities are already encoded in the agent’s memory, an increase in experimentation does not provide a commensurate improvement in the best-known state. As the agents gradually discover superior options, this initial shock of debiasing fades out and the performance of the debiased search ultimately converges to that of the continuously unbiased search.

In contrast, rebiasing leads to a second-order advantage. That is, after an initial drop in performance, rebiasing produces a temporary, but significant improvement in performance. A greater focus on the underexplored subset of the possibilities allows for a speeded accumulation of knowledge, which soon approaches that of the continuously unbiased search. As this happens, the implicit relative cost of experimentation declines and the agent takes advantage of the new bias. We call this effect a second-order advantage because it builds on the asymmetries in knowledge accumulation that were generated in the course of exercising the initial automatic bias.

### The optimal timing of rebiasing

Significant declines in relative performance may naturally cause the species and by extension their behaviors to go extinct, or the company to become bankrupt. However, if the challenge of survival is taken out of the picture, the net effect of volatility is not clear. In particular, short-term losses can be seen as a form of investment for delayed gains. With this in mind, we compare the levels of cumulative scores of various behaviors (biased, unbiased, debiased, and rebiased search) over different time spans. Note that there is no real-world time in the model. Therefore, as a proxy of actual time we take the count of search iterations or steps. In other words, one iteration of generating and evaluating a pair of alternatives corresponds to one unit on the time scale.

**FIGURE 6:**
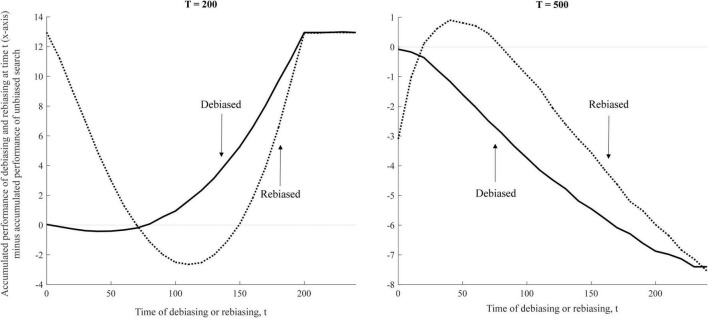
Accumulated performance of rebiasing and debiasing over a period of time T.

The curves in Figure 6 plot the relative cumulative performance of a given manipulation of biases. The value of zero indicates that the average accumulated performance of the unbiased and rebiased or debiased agents are equal. For example, a point on the solid black line (left panel) that coordinates approximately (50, 2.5) means that rebiasing at *t* = 50 in a setting with significant time pressure leads to the overall gain of approximately 2.5 performance scores over the entire period (*T* = 200).

Figure 6 shows that rebiasing (and not debiasing) can be a superior intervention. With short or moderate time spans in a given setting (*T* = 500), agents benefit from periodically changing their biases. In other words, if human decision makers have a sufficiently limited time to solve a certain recombination problem, i.e., if they have relatively few trial attempts, rebiased search may be their optimal form of behavior.

Strikingly, although debiasing occasionally outperforms rebiasing, it is never the dominant approach. Debiasing is always dominated either by continuously unbiased or by rebiased search. When it comes to recombination problems that involve active trial and errors, organizations should not seek to debias their decision makers. In fact, they may want to do the exact opposite and seek to rebias organizational decisions. This observation, unique to the present research, has important implications for how we manage human biases that originate in our less deliberate cognitive processes.

## Discussion

System 1 automatic evaluations are endemic to human mental functioning, and as some have argued may contribute to our intelligence. Yet because of them, our specific judgments are often deeply biased. Arbitrary signals activate our automatic preferences and make us gravitate toward some options even before we know how good or bad they truly are. This tendency may undermine the quality of any single choice. At the same time, it is so fast and effortless that over populations of choices it may prove to be useful and adaptive (e.g., Gigerenzer and Goldstein, 1996; Gigerenzer and Todd, 1999; Bernardo and Welch, 2001; Johnson and Fowler, 2011). Drawing on this prior work, we find that biases improve decision maker’s performance over a sequence of choices. As we illustrate, System 1 biases serve as a cognitive tool regulating excess experimentation, producing substantial benefits. Strikingly, this benefit of bias occurs even when there is no correlation between the variable of interest and the bias-generating variable. Automatic biases should be even more useful, and return value for longer, when they map closely onto environmental regularities (Gigerenzer and Todd, 1999).

In and of itself, this effect parallels other evolutionary advantages. But when paired with our present-day self-awareness and psychological toolkit, it offers the possibility of uncovering value beyond that of survival. Changing a bias, including debiasing, comes with a major short-term penalty: there is an immediate and profound decline in expected performance. However, the immediate disadvantage of changing biases are outweighed by the long-run benefits. Contrary to what might be anticipated, we find that organizations can most benefit by periodically reversing the biases of their decision makers. In complex settings with limited available time, a dominant strategy can be to rebias, in other words to strategically shift the overall decision making bias to its precise opposite. This provides a novel perspective on managing biases as previous work in experimental settings has focused almost exclusively on debiasing: in other words the reduction, correction, and elimination of bias (e.g., Wilson and Brekke, 1994; Wilson et al., 2000). The present analyses identify rebiasing as an unconsidered but highly effective strategy for organizations. The benefits of rebiasing, however, emerge only if decision makers reverse their biases at a calculated moment in time, when the benefits of the initial automatic preference are no longer materializing.

Time is an essential variable in our analyses. First, we use time to show that biases in solving recombination problems that involve active trial and error are not uniformly negative or positive. In complex environments full of uncertainty, acting on automatic preferences is associated with short-term gains in performance and yet long-term costs. In addition, time can underlie an important variance in how effectively organizations manage biases. We show that biases should be managed, and time is a critical component in the effectiveness of this process. The optimal strategy may be to first leverage initial biases, and then engage in a timely rebiasing, adopting the exact opposite automatic preference. Our work thus answers calls to explore the role of intuition and affect in decision making over time (see George and Dane, 2016). *Via* the computational experiments used in the present research, we can point to the plausibility of phenomena that would be otherwise difficult to observe empirically (e.g., Epstein, 1999; Gray et al., 2014; Jung et al., 2021; Schaller and Muthukrishna, 2021).

Although, we cannot say if the observed differences will translate into meaningful effects in the real world—this requires empirical measurement—within the modeled universe, the effects are not as small as they might seem. Indeed, the gain of biased search is ∼0.119, which is around 11%. Further, with regards to performance in highly competitive environments, even small differences can prove crucial. Seemingly minor discrepancies in outcomes accumulate over time (Hardy et al., 2022) and may provide key advantages over rivals, especially in winner take all competition formats. Consider a rivalry between two firms, in which company A achieving a certain market share will drive company B out of the market entirely and vice versa. In such a scenario, real-world differences far less than 11% could prove decisive.

A further important caveat concerns how the model time translates into the real-world time and whether such a translation is plausible. In other words, what is the meaning of 10, 100, or 1,000 search iterations in real-world settings? At this point, we cannot answer this question directly. But we can claim that a thousand iterations, or even more, may be well within many real-world time horizons over which performance plays out. To see this, consider the many decisions organizations make on a daily basis, i.e., decisions regarding personal remuneration, monetary and non-monetary rewards, product size, packaging, pricing, etc. All of these decisions seem to solve various problems and many of them take little to no time. At the same time, there is a combination of choices that will result in superior performance. Assuming that each possible combination of choices represents a single alternative in the model, by making day-to-day decisions, organizations effectively select different options. This means that a few years of routine organizational decision making can be realistically analogous to a thousand search iterations in the model. This, however, is only speculative at this point. Further empirical analyses of decision frequency in ecological contexts are needed to understand how the model time translates into the real-world time as well how organizations can use this to rebias productively.

Although judicious timing is clearly critical, another practical question is how feasible it is to debias or rebias decisions. Numerous experimental interventions have been developed in an effort to achieve unbiased or at least less biased decisions, with decidedly mixed success (Wilson and Brekke, 1994; Kahneman, 2003; Kahneman et al., 2011). Some interventions do attempt to push decision makers in the opposing direction, such as the consider-the-opposite strategy (Lord et al., 1984), or exhibiting pictures of widely admired Black Americans to reduce implicit prejudice (Dasgupta and Greenwald, 2001). However, the underlying goal is typically to shift decision makers toward neutrality, in other words to debias rather than rebias. For instance, Dasgupta and Greenwald (2001) presented White American research participants with photographs of Dr. Martin Luther King Jr. in the hopes of reducing their implicit preference for White over Black, not to create a bias against Whites. With regard to rebiasing at the individual level, there is the possibility of using framing to activate alternative automatic preferences (e.g., directly opposed values both endorsed by the same person, such as group loyalty vs. merit; Haidt, 2001; Chong and Druckman, 2007). A more pragmatic and sustainable option, readily available to most organizations, is to switch the key decision makers to persons already known to hold the opposite automatic inclinations. For example, an organization that senses it is no longer reaping the benefits of its initial automatic preferences and needs to re-bias might change their leadership team to executives with directly contrary automatic biases. Re-biasing, however, would not be advisable in cases where the initial bias maps closely on to environmental regularities, as often happens in the natural world (e.g., wild animals relying on predictive cues to identify predators and prey in their natural habitat). Yet, in the turbulent environments faced by many contemporary organizations, well-timed reversals in leadership approach could prove advantageous.

Consider an example of a football team. From the perspective of the coach, choosing the right players is a standard problem that requires trial and error. While searching for an efficient solution to this problem, the coach may automatically discard some options. For example, the coach may intuitively reject those alternatives that do not favor players with whom the coach has friendly relationships. However, should this coach be removed after a time, her or his successor is likely to already hold or shortly form a different pattern of liking and disliking toward the players. A change of the key decision maker, therefore, represents a basic instrument that can lead to a change in the automatic evaluations, or rebiasing, at the organizational level.

Our model indicates that the success of a debiasing or rebiasing intervention is contingent on intervening at the correct moment. But how can an individual or organization determine when that moment is, or in other words, where they are currently situated in the performance curve? We conjecture that an organization can leverage its traditional performance indicators to get a sense its performance has dropped substantially and is on a downward trajectory from earlier time periods relative to peers. If so, this suggests they could now benefit from a change in automatic decision tendencies at the top. Our results highlight to an organization that is underperforming relative to its comparative performance in the past, and decides they need a significant change, that rebiasing may benefit them more than debiasing.

Previous work has pointed to the possibly positive and adaptive role of biases (e.g., Gigerenzer and Todd, 1999; Johnson and Fowler, 2011). Building on this idea, we use simulations to capture the temporal dimension long under-recognized in the experimental literature. By doing so, we analyze the lifecycles of biases and demonstrate that time is an important factor in managing them. Notably, our longitudinal pattern is distinct, but also non-contradictory, to what scholars studying fast and frugal heuristics have previously theorized. Specifically, they suggest biases that lead to errors in one-shot laboratory experiments can be adaptive in the long term in complex naturalistic environments. In contrast, our simulations capture situations in which biases are beneficial in the short term but hurt performance in the long term—unless the decision making agent rebiases itself at an opportune moment. Although this argument is substantially different, it does not contradict the existing theories. Like Gigerenzer and colleagues, we argue that biases can be adaptive over multiple choices. However, we further suggest that this effect is non-monotone and may reverse over time. Organizations—unlike individuals—possess instruments to calibrate and manipulate biases, such as changing decision-making processes, redesigning organizational structures, or simply replacing key decision makers entirely (Christensen and Knudsen, 2010). That is, organizations have structural and contextual means to alter the effective biasedness of their decisions, and therefore can proactively and profitably manage their effects.

## Data availability statement

The complete data and code are posted on the Open Science Framework at https://osf.io/sypn2/?view_only=1b00c0d2dc964bafadf10215bfca4743.

## Author contributions

AK and EU ideated the project and wrote the manuscript. AK conceptualized the model and performed the analyses. Both authors contributed to the article and approved the submitted version.

## Footnotes

1 Combinations of these and similar decisions can be seen as locales on a rugged performance landscape (e.g., Levinthal, 1997; Rivkin, 2000). The idea in this line of work is simple: every (organizational) state is described as a collection of policies. States that differ by few policies are close to each other, whereas states that differ by many policies are distant. Naturally, correlation of performance tends to be higher for those states that are closer to each other and lower for those states that are far apart. On such a landscape, organizations tend to search within an immediate vicinity of the current state (see Simon, 1956; Levinthal, 1997). Our results are robust to such local adaptation on rugged performance landscapes simulated by means of the NK model (Kauffman and Levin, 1987; Kauffman, 1993; Rivkin, 2000).

2 Recall that *f*(*s*_*n*_) ∼ *N*(0, 1).
